# Influence of User Profile Attributes on e-Cigarette–Related Searches on YouTube: Machine Learning Clustering and Classification

**DOI:** 10.2196/42218

**Published:** 2023-04-12

**Authors:** Dhiraj Murthy, Juhan Lee, Hassan Dashtian, Grace Kong

**Affiliations:** 1 Computational Media Lab, School of Journalism and Media Moody College of Communication The University of Texas at Austin Austin, TX United States; 2 Department of Psychiatry Yale School of Medicine New Haven, CT United States

**Keywords:** electronic cigarettes, electronic nicotine delivery systems, ENDS, tobacco products, YouTube, social media, minority groups, exposure, youth, behavior, user, machine learning, policy

## Abstract

**Background:**

The proliferation of e-cigarette content on YouTube is concerning because of its possible effect on youth use behaviors. YouTube has a personalized search and recommendation algorithm that derives attributes from a user’s profile, such as age and sex. However, little is known about whether e-cigarette content is shown differently based on user characteristics.

**Objective:**

The aim of this study was to understand the influence of age and sex attributes of user profiles on e-cigarette–related YouTube search results.

**Methods:**

We created 16 fictitious YouTube profiles with ages of 16 and 24 years, sex (female and male), and ethnicity/race to search for 18 e-cigarette–related search terms. We used unsupervised (k-means clustering and classification) and supervised (graph convolutional network) machine learning and network analysis to characterize the variation in the search results of each profile. We further examined whether user attributes may play a role in e-cigarette–related content exposure by using networks and degree centrality.

**Results:**

We analyzed 4201 nonduplicate videos. Our k-means clustering suggested that the videos could be clustered into 3 categories. The graph convolutional network achieved high accuracy (0.72). Videos were classified based on content into 4 categories: product review (49.3%), health information (15.1%), instruction (26.9%), and other (8.5%). Underage users were exposed mostly to instructional videos (37.5%), with some indication that more female 16-year-old profiles were exposed to this content, while young adult age groups (24 years) were exposed mostly to product review videos (39.2%).

**Conclusions:**

Our results indicate that demographic attributes factor into YouTube’s algorithmic systems in the context of e-cigarette–related queries on YouTube. Specifically, differences in the age and sex attributes of user profiles do result in variance in both the videos presented in YouTube search results as well as in the types of these videos. We find that underage profiles were exposed to e-cigarette content despite YouTube’s age-restriction policy that ostensibly prohibits certain e-cigarette content. Greater enforcement of policies to restrict youth access to e-cigarette content is needed.

## Introduction

Nicotine exposure through e-cigarettes, particularly during adolescence, poses negative health outcomes such as brain maldevelopment and subsequent substance use [1]. In 2022, 9.4% (representing 2,550,000 students) of US middle and high school students reported using e-cigarettes in the past 30 days [1]. e-Cigarettes are also popular among adults (5.1% used them in the past 30 days in 2020), but these are most often used by young adults (15.6%) [2,3]. e-Cigarette use among adolescents and young adults (referred to as “youth” from here onward) may be driven, in part, by its heavy presence and positive portrayal on social media [4,5]. There is accumulating literature documenting e-cigarette promotion on social media. e-Cigarettes are portrayed on social media as fashionable, acceptable, and cool [6]. There are also themes that specifically appeal to youth, such as cartoon-based images on Instagram [7] and vape tricks (ie, blowing large vapor clouds or shapes like rings) on YouTube [8]. Studies have suggested that positive perceptions such as e-cigarette use being socially acceptable is related to its use among youth [9,10]. Studies have also shown that such positive portrayals of e-cigarettes on social media platforms have contributed to youth appeal and use behaviors [11]. For example, Lee et al [12] used state-level population data and found that the daily use of social media platforms, namely, Instagram, Snapchat, Facebook, and Twitter, was associated with e-cigarette use among adolescents, suggesting that youth may be exposed to e-cigarette–related information on social media. Given the high rate of social media usage by youth [13] and the unregulated environment [14], surveillance of e-cigarette–related content on social media platforms is warranted.

Social media platforms custom-tailor content to user characteristics [15]. However, these algorithms are proprietary, and it is unclear how information regarding e-cigarettes is featured to youth users. In this study, we examined how user profile attributes (ie, age and sex) influence the e-cigarette content being shown to youth users on YouTube—an online video streaming social media platform that has more than 2 billion users and is viewed more than 1 billion hours/day [16]. Users can upload and watch videos on YouTube and interact with other users by posting comments, reacting to videos (like/dislike), sharing content, and subscribing to YouTube channels. YouTube was the most frequently used social media platform in 2021, with 81% of the social media users reporting having used the platform [17]. Moreover, YouTube is the most popular platform among youth [8].

e-Cigarette content is prolific on YouTube. For instance, Huang et al [18] analyzed 28,000 e-cigarette–related YouTube videos and found that those videos had received more than 100 million views, indicating high engagement by users [18]. Further, e-cigarettes are frequently positively portrayed on YouTube and pro–e-cigarette videos are commonly sponsored by the e-cigarette industry [19]; 85% of the e-cigarette–related videos on YouTube are sponsored by e-cigarette marketers, including e-cigarette companies or people endorsing e-cigarette companies [20]. Pro–e-cigarette videos include portrayals of e-cigarettes as safer, cleaner, and less malodorous than combustible cigarettes [21]; videos showcasing or teaching how to conduct vape tricks (ie, using e-cigarettes to blow large, thick amounts of exhaled aerosol or shapes) [8]; modification of e-cigarette devices for unintended purposes such as increasing the temperature and using other substances in it [19,22,23]; instructions on how to use e-cigarettes (eg, how to puff) [24]; product reviews [25]; and health information or misinformation about e-cigarette use [26]. Concerningly, these e-cigarette contents are readily available on YouTube without a warning label/statement [27], and these videos are easily accessible to youth [4]. In sum, there are abundant e-cigarette–related videos on YouTube. However, less known is specifically what content youth are exposed to. All users do not receive the same results when they search for the same terms on YouTube. This is partially due to YouTube’s personalized search and recommendation algorithms, which consider, to some extent, a user’s age, sex, and the history of the searched items by that specific user [28,29].

YouTube’s search and recommendation algorithms are responsible for creating personalized content for users from an ever-growing collection of videos. Similar to other social networks, YouTube has undergone a paradigm shift toward using deep machine learning—systems based on artificial neural networks—as a solution for scaling the systems used by YouTube’s search and recommendation algorithms [30]. However, the opaque nature of the search and recommendation algorithms poses questions concerning whether algorithmic visibility can be evaluated. Search and recommendation algorithms may be developed to take viewers’ demographic profiles (eg, age, sex) as inputs in determining what search results users receive. Therefore, YouTube’s search and recommendation algorithms have important public health implications. For instance, males have consistently shown a higher level of e-cigarette use among adolescents and adults [31], and data suggest that e-cigarette–related videos such as vape tricks videos mostly feature young men and seem to be targeting this population [8]. A recent study identified that e-cigarette content on YouTube contained themes related to product reviews (provide reviews of e-cigarette products), instructional videos (teach viewers how to use, modify, or create e-cigarette products), health information (provide health information related to e-cigarettes), vape tricks (feature different vape tricks), cannabis (cannabis vaping–related topics), and other (a variety of other themes such as news clips related to e-cigarette use) [19]. However, less known is whether these video themes are differentially exposed to users by their demographic attributes. Such information is important to inform tobacco regulatory actions in restricting marketing that targets at-risk populations such as underage youth, and it can be used to inform how prevention strategies such as countermarketing can be targeted to these populations.

## Methods

### Overview

**Figure 1:**
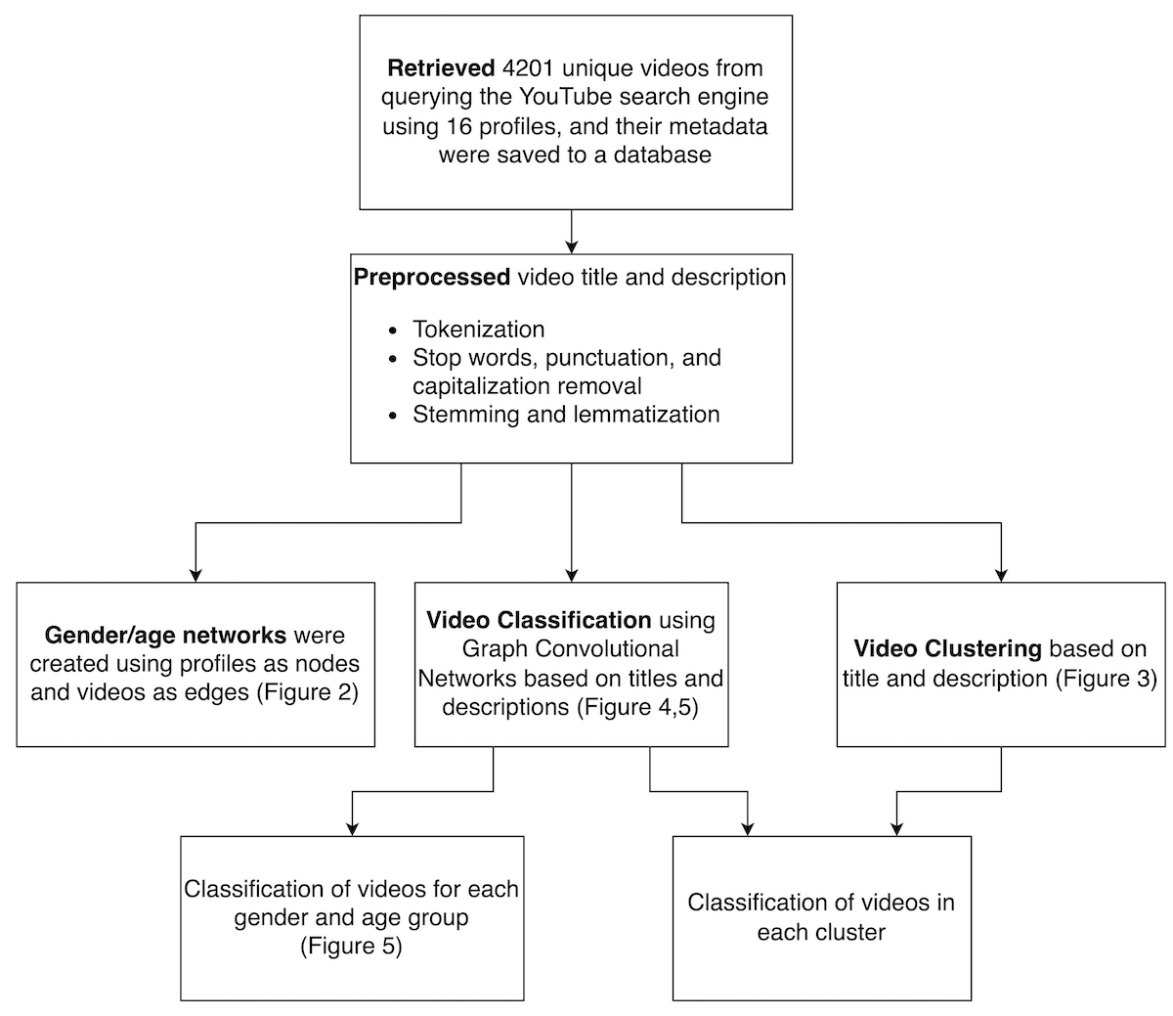
Overall framework of data collection, preprocessing, and analytics.

The goal of this study was to understand the role of the demographic factors (ie, sex, age) of YouTube users’ profiles in influencing the variations in e-cigarette–related search results presented to users. To accomplish this goal, we developed a 3-step framework, which combined computational methods and human labeling (Figure 1). First, we used an unsupervised machine learning method, the k-means method, which used the distribution of words in the video data (ie, titles and descriptions) to cluster the videos into themes. Human-labeled data sets using titles and descriptions of the YouTube videos were then used to confirm the themes identified in our k-means clustering results. Second, we used this human-labeled data set to train a supervised machine learning method, that is, the graph convolutional network (GCN), to classify all the videos in our data set based on their identified themes. Finally, we performed unsupervised network analysis to measure how YouTube video results varied by user attributes (ie, age and sex). We examined whether there were differences in the video themes between different age and sex profiles. The application of these machine learning–based methods is novel in tobacco regulatory science work using social media data. Our approach is also scalable to large volumes of data and can be extended to a variety of social media platforms.

### Ethical Considerations

This research is not deemed as human subjects research according to the definition provided by the Office of Human Research Protections, US Department of Health and Human Services. We examined publicly available data, and we did not report any identifying information of the content observed on social media. Additionally, this observational study was deemed exempt as human subjects research by the Yale Institutional Review Board (HIC 2000028350).

### Search Methods

We created 16 fictitious profiles on YouTube that sought to vary and reflect particular demographic attributes (ie, age, sex, and race) [32]. Profile photos were not added. To attempt to reflect particular racial and ethnic attributes, we created profiles by using common African American, Hispanic, and White first and last names by using existing name data [33]. The profiles consisted of African American females and males aged 16 and 24 years (4 profiles), Hispanic females and males aged 16 and 24 years (4 profiles), and 2 sets of White females and males aged 16 and 24 years (8 profiles). We oversampled White users to be more reflective of the e-cigarette use population. To create each fictitious profile on YouTube, we used a new SIM card and phone number and performed a factory reset of an Android phone. Sex and age were entered during each fictitious account creation. No other demographic metadata were included during account creation. We used a mobile phone rather than a web browser to conduct our searches to best replicate how youth access YouTube content [13].

During the course of a week in June 2020, we collected data for 2-3 profiles per day. Once we collected 140 videos per profile per search term, we factory reset the Android phone and moved to the next profile. For each profile, the following keywords were searched for each profile by using Orbot, a mobile app that allows one to use an anonymized Tor bridge (to avoid location or IP address personalization): box mods, cigalikes, disposable e-cigs, disposables, disposable vape, e-cig, e-cigarette, e-juice, electronic cigarette, e-liquid, ENDS, pod mods, vape, vaping, vape juice, vape mods, vape pens, vape pods. Studies typically examine the first page [18,20,34] of the search results on YouTube, which has 20 videos, or the first 2 pages, which has 40 videos. However, some users may search through multiple pages if they do not find what they are looking for in the first few pages. Thus, for each of our 16 fictitious YouTube profiles, we searched through 7 pages (140 videos) for each of our keywords (n=5875). This approach is therefore far more aggressive than previous work [35]. After removing duplicates (n=1674), we arrived at the final sample (N=4201) of unique videos. We collected video metadata such as title, description, transcript, view counts, likes/dislikes, comments, date published, channel name, and category. The methods are further explained in Dashtian et al [32].

### Preprocessing Data

We converted the text into numerical form so that we can apply machine learning algorithms such as clustering and classification to the data. The preprocessing steps included tokenization, stop words removal, stemming, and lemmatization. Tokenization is the process of splitting a set of texts into words (also called tokens) and then removing certain characters such as blank sequences and punctuation. Stop words are usually frequent in English text (eg, a, an, the, that, I, be, other, with). The goal of both stemming and lemmatization is to find the base form of a word from its inflectional forms and derivatives (eg, vaped, vaping have a base of vape). We used Porter stemmer, an algorithm which has been successfully used by others for the stemming of health-related texts for machine learning purposes [36].

### Video Clustering (Unsupervised Machine Learning)

K-means automatically arranges texts into clusters such that text data within clusters are relatively similar in terms of content when compared to text data in other clusters [37]. Another health-related work [38] has successfully used the k-means clustering algorithm for automated text classification. We therefore chose to use k-means to categorize video types. In our case, the input to the k-means clustering is preprocessed text (video title and the description provided by the uploader to describe the video). We used the elbow method to find the optimum value for the number of clusters (k). The elbow method provides a good indication that the underlying model and number of (k) fits best at that point and has been successfully used in other health-related machine learning studies [39]. We examined the results visually to discern the point at which diminishing returns are observed (ie, an elbow appears). K-means seeks to cluster around optimal centroids (ie, cluster centers). The best placement of initial centroid positions is a standard method for maximizing the k-means clustering process. To avoid any bias, we randomly selected initial centroids and iterated the algorithm several times for each k to confirm that the initial centroids do not change our optimized clustering results. We measured cosine similarity to generate a measure of similarity between each video and the other videos in the search results. Cosine similarity is a measure mostly used for k-means clustering of text documents. The distance matrix was then converted into a 2D array by using multidimensional scaling.

### Video Classification (Human Labeling)

Members of the research team with expertise in e-cigarettes randomly selected videos from the full corpus of the collected videos (n=1000) [19] and labeled the videos by the following classes: (1) product review (ie, an individual(s) providing a review of an e-cigarette product), (2) health information (ie, health information related to e-cigarette use), (3) instructional (ie, a tutorial on how to use an e-cigarette or how to modify an e-cigarette), and (4) other (which consists of a variety of other themes, including cannabis, television/news clips, vape tricks). Interrater reliability (Cohen κ) was 0.93, indicating “almost perfect” agreement between coders. These categories were used in previous research [32]. Please refer to Kong et al [19] for more information on how these themes were determined and labeled.

### Text Classification Using GCNs (Supervised Machine Learning)

We used GCN, which is a supervised machine learning method, to classify data (ie, titles and descriptions) by theme to better understand the unique clusters identified through k-means clustering. In GCN, word frequency and word co-occurrence information are used to build the word-to-word and word-to-video edges (ie, as common videos between pairs), respectively. We also classified the nodes (ie, entities in the network) instead of the actual videos. The entities in the network represented just the nodes in the graph. These do not refer to the themes. GCN has shown strong performance for classification with a small portion of labeled data similar to the data used in our study [40].

To model the global word co-occurrence, we built a large 2-mode graph (ie, 2 types of nodes). Our graph contains word nodes (which represent single words) and document nodes (which represent whole documents with many words). See Multimedia Appendix 1 for a visual rendering of the relationship between the document nodes and word nodes. Specifically, the first mode of nodes consists of words and the second mode of nodes consists of documents with titles and descriptions (ie, with many words). One document represents 1 video (title and description together). Document nodes and word nodes are interconnected and intraconnected. The number of nodes in the text graph |V| is the number of documents (document nodes) plus the number of unique words in the documents (word nodes). We set feature matrix X = I as an identity matrix, which means every word or document is represented as a 1-hot vector as input to text GCN. One-hot encoding converts categorical data into binary values suitable for machine learning algorithms. We build edges (ie, connections) between nodes based on word occurrence in documents (document-word edges) and word co-occurrence in the whole corpus (word-word edges). The weight of the edge between a document node and a word node is the term frequency-inverse document frequency of the word in the document. Term frequency is the number of times the word appears in the document, and inverse document frequency is the logarithmically scaled inverse fraction of the number of documents that contain the word. After performing clustering and classification on preprocessed data, we calculated the percentage of each video type (derived from classification) in each category (derived from clustering).

### Profiles Network

The frequency of common videos between different ages and sexes can be used as a measure to quantify the strength of the relationships between these variables. For example, the overlap of videos among the same sex and age profiles can be used to discern whether users with these attributes (eg, both female and male, adolescents or young adults) receive similar information from YouTube’s search engine. Furthermore, the connections between nodes in a network provide information about the structure of the network. We can also use the number of connections of a node in each demographic group to identify the most influential nodes in the network. Specifically, the network of 4 demographic groups can be represented as nodes with their edges representing common videos between pairs of groups. To show the connections, we plotted a line between two groups and calculated the number of common videos between them. Lines with a larger value represent more common videos between a pair than lines with smaller values. We assessed 2 separate networks: one with common videos between age and sex and another that assessed a combination of the two.

## Results

### Video Clustering (Unsupervised Machine Learning)

**Figure 2:**
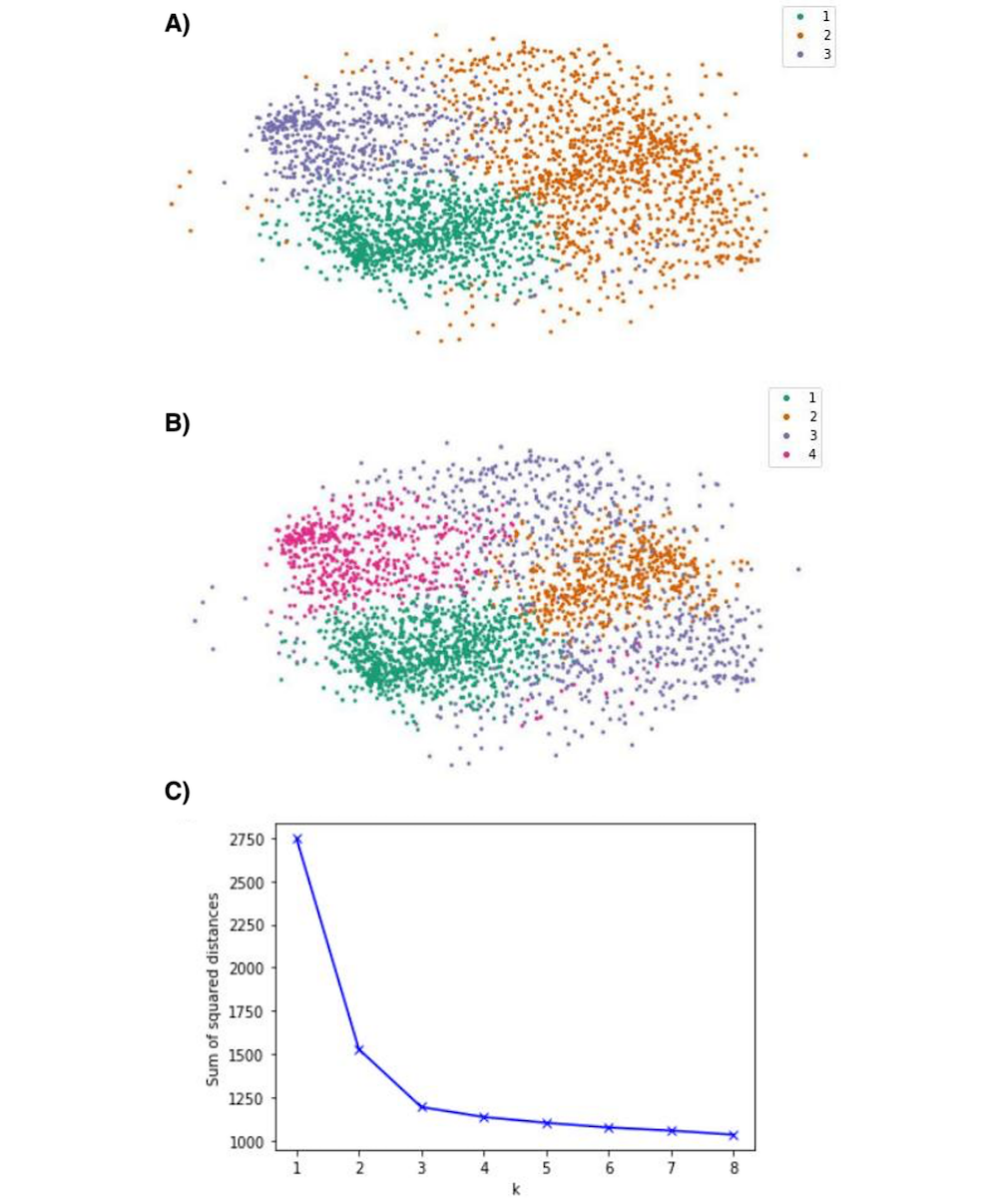
Each dot in (A)/(B) indicates a video and each color represents a cluster. (A) Videos categorized into 3 clusters. (B) Videos categorized into 4 clusters. (C) Elbow method results, which show the sum of the squared distances as a function of the number of clusters (k).

To better understand which content shows up for different demographic profiles, we identified the types of videos in our data set by using k-means to cluster videos. Figures 2A and B illustrate the video clusters as 3 clusters and 4 clusters, respectively. The former had 3 distinct topical clusters, whereas the latter had 3 distinct topical clusters and 1 diffuse cluster (that likely represents the “other” content cluster). The elbow method indicated that the plateau (ie, the first stable k value in the sum of squared distances) is at k=3 (Figure 2C). In some cases, the elbow method has ambiguity [41]. However, in our case, we had a clear result that videos can be automatically clustered into 3 main clusters.

### Video Classification (Human Labeling)

**Figure 3:**
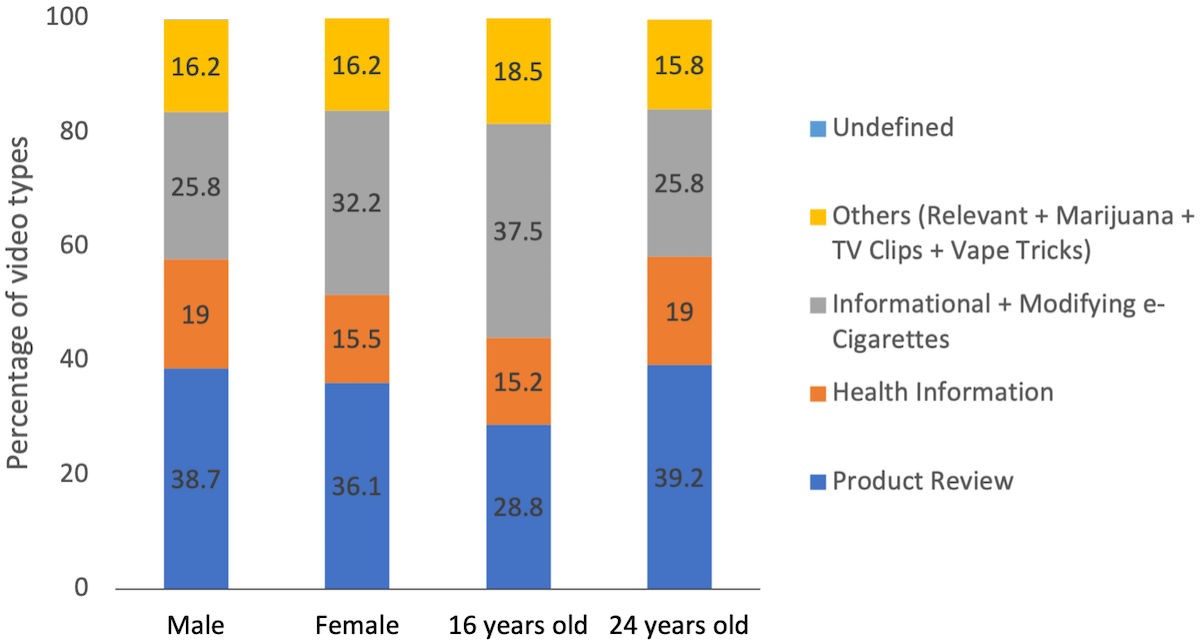
Prevalence of video type shown, split by demographic variables. The percentage of each label (class) is shown based on the results from graph convolutional networks. TV: television.

**Figure 4:**
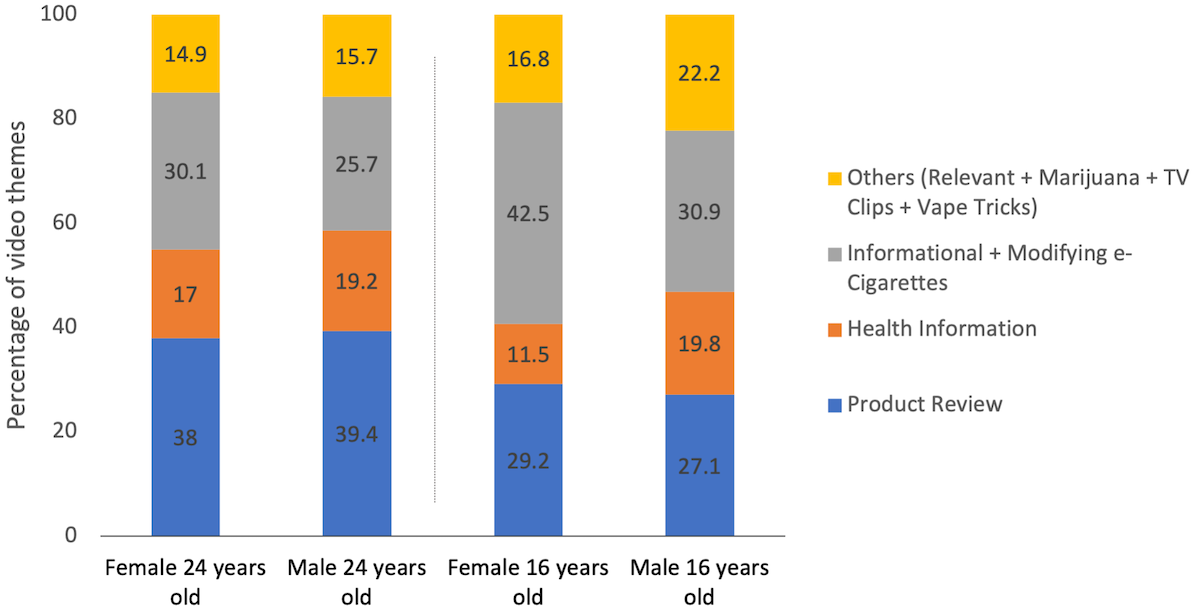
Results of the classification of videos in each demographic group. We grouped YouTube profiles based on age (24 or 16 years old) and sex (male and female). TV: television.

Human labeling identified 3 distinct classes: (1) product reviews, (2) instructional, and (3) health information. We also included a fourth catchall class of “others” for any videos that did not fit into the other 3 distinct classes. Product reviews are videos that provide reviews of e-cigarette products, instructional videos provide instructions on how to use/modify/create e-cigarette products, health information videos provide information on the health risk of e-cigarettes, and other videos are topics that do not fall into these 3 classes and include a range of topics such as cannabis and vape tricks. We found that GCN was able to successfully classify videos based on these 3 distinct classes as well as a separate “other” class. Overall, product review was the most common type of videos identified (49.3%), followed by instructional (26.9%), health information (15.1%), and other (eg, cannabis, television/news clip) (8.5%). We further estimated the prevalence of each video type exposure by demographic attributes (Figure 3). For all demographic groups, except the 16-year-old group, product review videos showed the highest percentage in the search results, followed by instructional videos. Instructional videos showed the highest percentage in the search results of 16-year-old students. We estimated the prevalence of video themes separated by age and sex (Figure 4). The product review label was the dominant class for 24-year-old male (39.4%) and 24-year-old female (38%) profiles. Instructional videos showed the highest percentage in the search results of 16-year-old female (42.5%) and 16-year-old male (30.9%) profiles; notably, the 16-year-old female profile had the highest percentage of search results for this label. All profiles were least exposed to health information videos.

### Text Classification Using GCNs (Supervised Machine Learning)

We used text classification using GCN, a supervised machine learning technique, to classify the text of video titles and description into human-labeled classes (ie, product review, health information, instructional, other). We found that the accuracy of the GCN model for the classification of e-cigarette–related YouTube videos is 0.72 for the parameters that we set. The precision, recall, and F1-score values were 0.70, 0.78, and 0.74, respectively.

### Profiles Network

**Figure 5:**
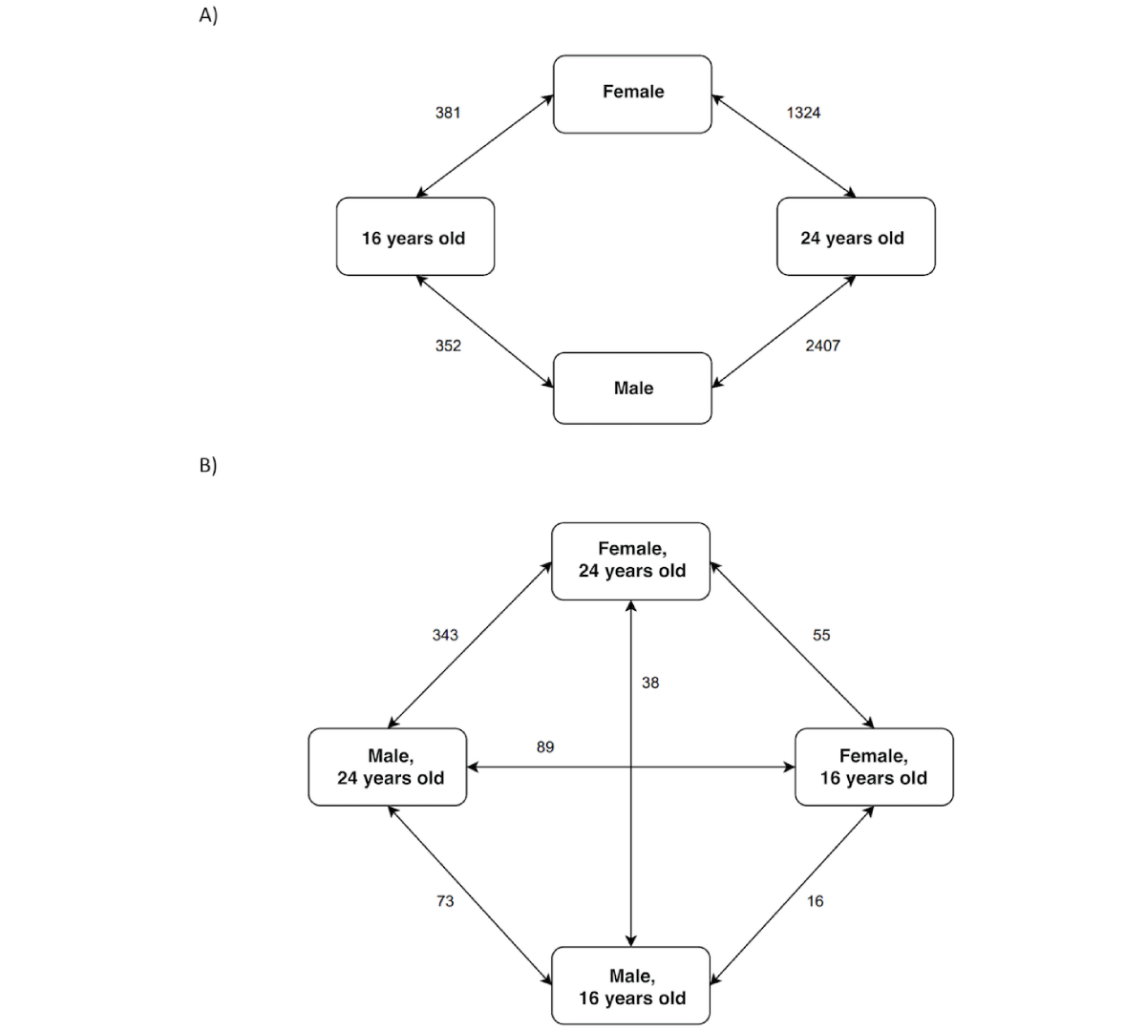
Network of demographic attributes and videos. Edge weights are provided next to the edge line between 2 pairs, and these edge weight values indicate the number of common videos between 2 corresponding nodes (ie, between the demographic attributes of sex and age).

The connections between the profile groups based on the common videos that were retrieved from the YouTube search are shown in Figure 5. The number of common edges between 16-year-old and 24-year-old pairs was the lowest among the other pairs. As shown in Figure 5A, the connection between the nodes of 24 years old and male is very strong, as indicated by the edge weight of n=2407 (ie, the number of common videos). We also constructed another network by using a combination of age and sex. The videos of all the profiles were grouped into 4 subsets: 24-year-old male, 16-year-old male, 24-year-old female, and 16-year-old female. Similar to that in the previous network, each node in the network represents one of these groups, and common videos between pairs of groups are shown as an edge. Compared to the previous network (Figure 5A), the network of combined age and sex (Figure 5B) had fewer edges (connections). When we examined the network of age and sex together, we imposed further restrictions on the videos that belonged to a specific node. Thus, the number of videos and therefore, the number of connections between nodes in the network of age and sex was smaller than that of age or sex alone. Figure 5B shows that 24-year-old male and 24-year-old female profiles have the highest number of common edges, while 16-year-old male and 16-year-old female profiles have the lowest number of common edges.

## Discussion

### Principal Findings

In this study, we examined how YouTube profile attributes, specifically age and sex, affected e-cigarette–related YouTube search results. Our profile network analysis indicated that there were more common videos between male and female 24-year-old profiles relative to other demographic groupings. Using our own human-labeled data, we developed a GCN machine learning model that was able to classify the videos into 4 main classes. We found that the highest proportion of younger age groups (16 years old) was exposed to instructional videos (37.5%), while the highest proportion of young adult age groups (24 years old) were exposed to product review videos (39.2%). Additionally, the group with the highest proportion of exposure to instructional videos was 16-year-old females relative to other age/sex pairs. Our findings are consistent with prior studies that observed that common video themes related to e-cigarettes on YouTube were product reviews and instructional videos on how to use/modify/create videos [8,19,22,25]. However, our results uniquely contribute to the literature by demonstrating that demographic attributes factor into YouTube’s algorithmic systems in how video themes are differentially shown to profiles with different age/gender attributes.

It is unclear what drives the differences in exposure to e-cigarette content and the volume of this content among different demographic profiles. Previous studies have shown that age and sex affect the results delivered in search engines (eg, Google) [15]. Our findings are consistent with other research that indicate that YouTube also may use demographic information to provide the most relevant information to users [29]. Specifically, Hussein et al [29] found that once a user develops a watch history in the YouTube search engine, the demographic attributes do affect the extent of content recommended to them. However, in this study, we used the same search words between each profile and used a mobile phone that was factory reset after each profile’s searches were conducted to prevent tailoring of search results. It is therefore unlikely that these factors account for differences in exposure to e-cigarette content. It appears that YouTube’s search engines and recommendation algorithms are driven by the demographic factors of its users. Personalization of search engines, where individual users receive distinct results for the same search query, has also led to public concerns about the so-called “filter bubble” effects [42], where users are unable to access diverse information that a search engine’s algorithm decides is irrelevant to a user [43]. Our results indicate that there might be differences in the type of exposure specific to e-cigarettes that are provided to different demographic groups. We further break down these differences in terms of age and sex attributes.

Our network of search results, which shows the influence of age and sex on search results, indicates a noteworthy difference between the number of edges (common videos) for various pairs of nodes (common videos between 2 groups) in the network, including male/female and 16-year-old and 24-year-old profiles. For example, the videos common to both 16-year-old and 24-year-old groups are the lowest. However, the second network analysis showed that 24-year-old male group and 24-year-old female group pairs have the highest number of common videos. There is a greater number of edges between the male group and 24-year-old group than between the female and the 24-year-old group, indicating that males and 24-year-old groups have more common videos than females and 24-year-old groups. These results indicate that 24-year-old profiles are most exposed to e-cigarette content, and this exposure is greater among 24-year-old male groups compared to their female counterparts.

Our finding that e-cigarette content is mostly available to male young adult groups is consistent with research findings that show that e-cigarette–related videos on YouTube feature more males. For instance, an examination of vape tricks on YouTube showed that 80% of the vape tricks videos featured young adult males [8]. There is also research showing that males are more engaged with YouTube content than females. Khan [44] found that male users are more likely to read comments on YouTube; Molyneaux et al [45] found that there was a greater number of comments posted by male users. Perhaps, the high engagement of males on social media platforms such as YouTube can explain the higher e-cigarette use rates among males. A review on e-cigarette use behaviors among adolescents showed that e-cigarettes are used more by male adolescents than by female adolescents [46], and national data also show that e-cigarette use is higher among male adolescents and young adults [47]. However, it is important to also highlight that e-cigarette use among females is also high: up to 20% of females in middle and high school surveyed in a study in 2020 were found to use e-cigarettes [47]. It is possible that females are engaging with e-cigarette–related social media content but doing so differently from males. For instance, there was no difference between males and females in viewing YouTube videos [44] or in the rating of YouTube videos [45], suggesting that females are engaging with YouTube content similarly as males.

The lower number of e-cigarette–related videos shown to 16-year-old profiles than 24-year-old profiles may be due, in part, to the age-restriction process of e-cigarette–related content by YouTube. YouTube’s current policy prohibits tobacco-related advertisements. YouTube considers content that “promotes a product that contains drugs, nicotine …” as age-restricted content [16]. They exemplified “a video reviewing brands of nicotine e-liquid” as an example of age-restricted content. This rule may explain why we observed more product review videos in the 24-year-old group (39.2%) compared to those in the 16-year-old group (28.8%). This finding also suggests that despite these self-imposed limits on e-cigarette promotional content on YouTube, there is evidence that these restrictions may be loosely implemented and content that are restricted may be shown to underage minors on this and other social media platforms [14,19,48]. It is noteworthy that in our study, the 16-year-old profiles were exposed to e-cigarette content despite YouTube’s age-restriction policy that prohibits certain e-cigarette content such as product reviews. This finding is consistent with that in other studies that found that e-cigarette content such as vape tricks were readily available using non–age-verified accounts [8]. This study highlights the importance of strong policies and the enforcement of these policies to prohibit the exposure of e-cigarette–related videos to youth on YouTube. This finding also suggests that young adults are the highest consumers of e-cigarettes among adults [49]; they may search for more information about e-cigarette products to purchase through product reviews.

Concerningly, the instruction label was observed in the highest percentage (37.5%) of search results of the 16-year-old group, and exposure to instructional videos among 16-year-old female profiles was particularly high (42.5%), suggesting that underage youth are more exposed to instructional videos, which may provide tutorials on e-cigarette use. Further, instructional videos include other content such as how to hack or modify the device to use for unintended purpose as well as to use cannabis [22,23]. The high prevalence of modification of e-cigarette content on YouTube has been shown in other studies. For instance, Massey et al [23] analyzed 168 e-cigarette–related YouTube videos and found that 20.2% of the videos were modifications of e-liquids to using cannabis. Future studies should identify whether youth modify/hack e-cigarettes and the health implications of engaging in such behaviors.

### Future Work and Limitations

Several limitations in this study are noteworthy. First, we might have missed potential search terms related to e-cigarettes. For example, these may include brand-specific terms (eg, Juuling) and e-cigarette use–related slang (eg, stick). Thus, our collected videos may not represent an exhaustive list of e-cigarette–related videos. However, our study uses 18 e-cigarette–related search terms that were successfully tested and used to collect a broad range of e-cigarette–related YouTube videos [32]. Second, due to a limited number of fictitious profiles, our findings do have limits in terms of generalizability. Third, we included race/ethnicity as an element when creating profiles (ie, White, African American, and Hispanic) to be inclusive of diverse racial backgrounds. The first and last names of each profile were randomly selected by choosing names from existing data sets that were shown to be most commonly associated with a specific race/ethnicity [33,50]. However, as we created a limited number of fictitious profiles, we did not have enough data points for each race/ethnicity to incorporate machine learning to determine whether search results differed by race/ethnicity. Fourth, we only used 2 age groups (ie, 16 and 24 years), and it is possible that the search results may be different if younger or older age groups were used. Future research should therefore place an emphasis on assessing whether race/ethnicity as well as other factors (eg, viewing history, age) has an effect on search results related to e-cigarettes on YouTube. Fifth, anonymous Tor-based IP addresses may have influenced our search results; therefore, results may differ if searches were to be conducted using nonanonymized IP addresses. There may be other factors that may drive results, such as the date/time of searches as well as what content is popular on YouTube at a given time. Sixth, we cannot confirm how, whether, or to what extent YouTube’s personalized search parameters read the demographic attributes (ie, age, sex, and race) that we populated our fictitious profiles with because the algorithm is proprietary. However, we used a factory-reset Android device without any search history or cookies to avoid any implicit bias in the results. The searches were conducted using the same terms to ensure that the differences between profiles, from our vantage point, are only the demographic characteristics. Nevertheless, as we only used search results collected from a mobile device, future work can explore whether web-based results are different. Seventh, we applied our methods, that is, natural language processing, video classification, and network modeling to only a single platform, that is, YouTube. Future studies would therefore benefit from extending our methodological framework to other social media platforms. Eighth, given that after we collected 140 videos per profile per search term, we factory reset our phone and moved to the next profile; our approach does not emulate or reflect the high levels of personalization that a user who uses YouTube everyday might experience. Future studies would therefore benefit by comparing our results from collecting data from YouTube in 1 setting with fictitious profile data collection done over a longer period and with some levels of variation. Ninth, we did not undertake statistical tests comparing the proportions of content classification by profile demographics nor were we able to determine how each theme was manifested by demographic attributes (eg, was health information present more for male profiles than female profiles?). Future work could make these comparisons based on the classes identified by the GCN analysis and determine how and why content themes vary by different profile attributes. Lastly, as we did not have a control group in our data collection methods, future work would benefit from the use of a control group and the examination of some of these variables.

### Conclusion

Our findings underscore the value of machine learning methods in studying how profile attributes on YouTube may influence e-cigarette–related content and move the field forward by highlighting the critical need to take into consideration how social media algorithms work in practice. We used unsupervised (k-means clustering) and supervised (GCN classification) machine learning models in combination with network models to study the variation of e-cigarette–related videos on YouTube. Our methods were designed to specifically identify the similarities and differences in the videos by using selected demographic attributes, that is, age and sex. Collectively, our results suggest that advanced computational methods can be used to help understand how YouTube’s current search and recommendation algorithm customizes e-cigarette–related content based on demographic attributes such as sex and age. This suggests an urgent need for surveillance and prohibition of e-cigarette–related content on social media such as YouTube to prevent e-cigarette use among youth.

## Appendix

Multimedia Appendix 1 Document node and word node composition and relationship.

## Abbreviations

GCN: graph convolutional network
