# Supplementary figures and images for: Influence of User Profile Attributes on e-Cigarette–Related Searches on YouTube: Machine Learning Clustering and Classification
Source: JMIR Infodemiology. 2023 Apr 12;3:e42218. doi: 10.2196/42218 (PMC10139687; doi:10.2196/42218)

**Multimedia Appendix**

**Appendix** 1**:** Document node and word node composition and relationship.


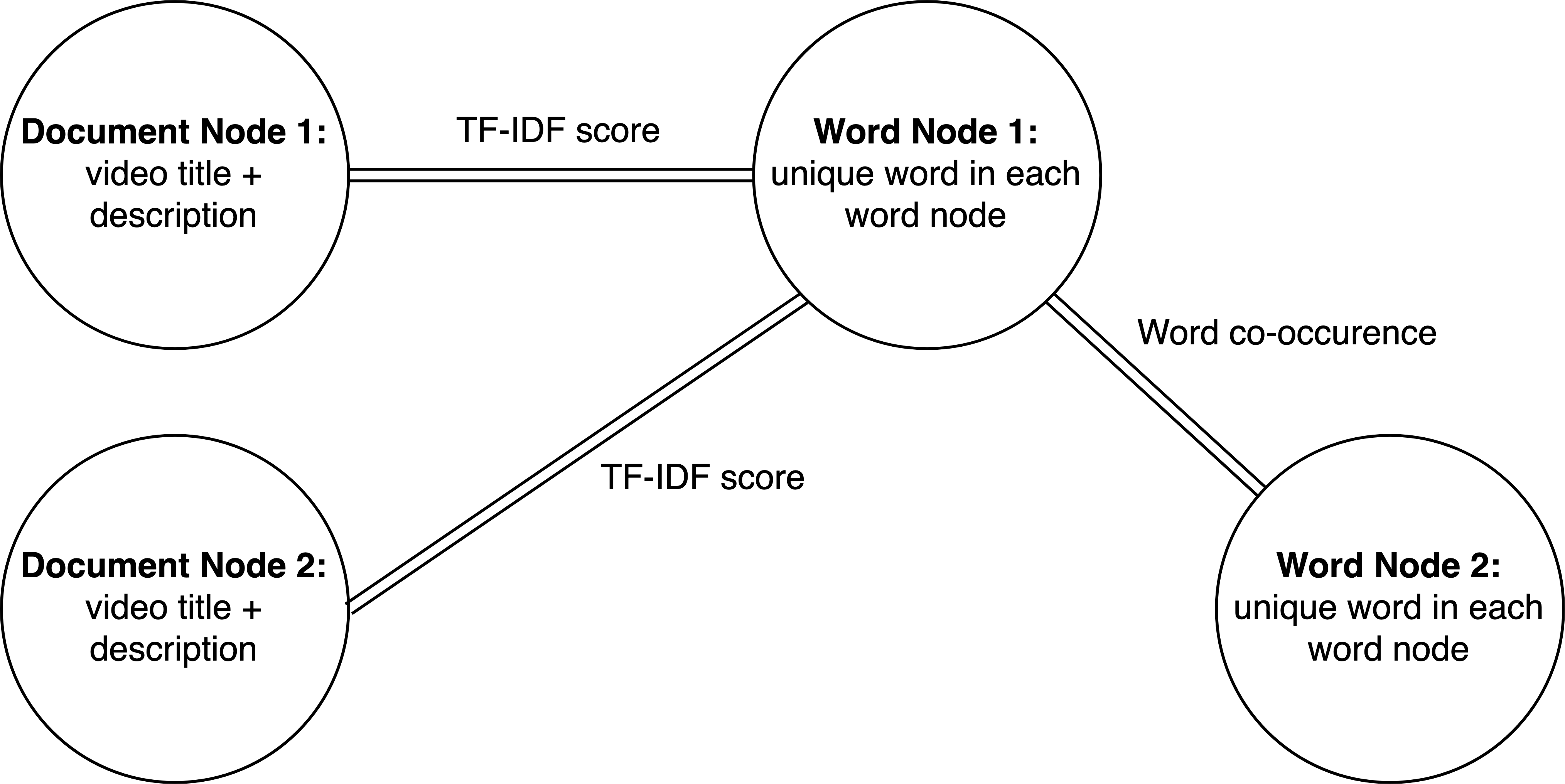

Supplement: Multimedia Appendix 1 [file infodemiology_v3i1e42218_app1.docx]
